# Conversion of Cannabidiol Following Oral Administration: Authors' Response to Grotenhermen et al. DOI: 10.1089/can.2016.0036

**DOI:** 10.1089/can.2016.0038

**Published:** 2017-01-01

**Authors:** Marcel O. Bonn-Miller, Stan L. Banks, Terri Sebree

**Affiliations:** ^1^ Department of Psychiatry, University of Pennsylvania Perelman School of Medicine, Philadelphia, Pennsylvania.; ^2^ Zynerba Pharmaceuticals, Inc., Devon, Pennsylvania.

**Keywords:** CBD, conversion, gastric, oral administration, THC

## Abstract

In a commentary, Grotenhermen, Russo, and Zuardi questioned not only the clinical relevance but also the conclusions of a recently published study by Merrick et al. on the conversion of cannabidiol (CBD) to delta-8 and delta-9-tetrahydrocannabinol (THC) in simulated gastric fluid. In response, this article aims to provide a thorough review of the *in vitro* and *in vivo* studies of gastric CBD conversion as well as potential consequences resulting from such conversion. Findings highlight (1) consistent evidence over the past half century of gastric conversion of CBD, (2) evidence from human studies, indicating the importance of testing for THC metabolites as well as a number of other cannabinoids in the detection of such conversion, and (3) THC-like effects after administration of oral CBD in humans that may not only stem from CBD's conversion to THC, but also its conversion to 9α-hydroxy-hexahydrocannabinol and 8α-hydroxy-*iso*-hexahydrocannabinol. These findings, coupled with a number of limitations in the existing literature, point to the need for large-scale human studies, specifically designed to explore gastric conversion and potential THC-like side effects after oral administration of CBD.

After publication of the article “Identification of psychoactive degradants of cannabidiol in simulated gastric and physiological fluid,”^1^ Grotenhermen, Russo, and Zuardi wrote a commentary arguing against the gastric conversion of CBD to THC in humans.^2^ The primary focus of the initial article^1^ was on gastric conversion of CBD to delta-8-THC and delta-9-THC, with a note of caution about potential adverse effects with unintended THC exposure. In their commentary, Grotenhermen et al. primarily take aim at the clinical implications of CBD conversion to THC, although they do end their article by raising doubt about whether the conversion itself occurs.^2^ In this rebuttal, we aim to address both the argument against conversion of CBD to THC and its clinical implications in humans.

First, it is important to note that the article by Merrick et al.^1^ was not the first study to identify conversion from CBD to THC in gastric fluid. Indeed, conversion of CBD to THC was described in 1968 by Gaoni and Mechoulam.^3^ Later, Harvey et al.^4^ found delta-9 THC, as well as a number of other cannabinoids, in human urine after daily administration of 600 mg doses of CBD. More recently, Watanabe et al.^5^ documented conversion from CBD to not only THC but also cannabinol, 9α-hydroxy-hexahydrocannabinol, and 8α-hydroxy-*iso*-hexahydrocannabinol (9α-OH-HHC and 8α-OH-*iso*-HHC) in simulated gastric juice. In fact, the consistent findings of CBD conversion to THC were highlighted in a recent review by Ujváry and Hanuš.^6^

Findings from the aforementioned studies are in stark contrast to Grotenhermen and colleagues' statement that “we have enough data to be reassured, that the acidic gastric environment during normal gastrointestinal transit DOES NOT ‘expose patients treated with oral CBD to levels of THC and other psychoactive cannabinoids.’”^2^ As it appears that their conclusion was based on data from two studies that examined conversion, we will take the opportunity to discuss these studies in depth.

The first cited study was conducted by Consroe et al.^7^ among a sample of eight men and six women with Huntington's disease. Participants were given 10 mg/kg/day of CBD (dissolved in sesame oil) and a sesame oil placebo for 6 weeks each in a cross-over design. Blood samples were obtained weekly for a total of 15 weeks and tested for CBD and THC (parent drug only, no metabolites). Although the authors found detectable levels of CBD, THC was not detected in the subjects' plasma.

The second, and most recent, study was conducted among 16 healthy volunteers who received 600 mg of CBD in a cross-over double-blind study.^8^ In their commentary, Grotenhermen and colleagues describe the study as showing no elevation of THC or its metabolites (i.e., 11-hydroxy-delta-9-tetrahydrocannabinol [11-OH-THC] or 11-nor-9-carboxy-delta-9-tetrahydrocannabinol [THC-COOH]) in plasma after administration of CBD. Upon close inspection of this article, however, one can see not only detected levels but also steady increases in 11-OH-THC and THC-COOH in the 3 h after acute administration of CBD (See Figure 6 in Ref.^8^). Note that 11-OH-THC is a psychoactive metabolite that has comparable and sometimes greater effects than THC.^9^ Unfortunately, it does not appear that Martin-Santos et al. tested statistically for differences in THC metabolites between placebo and CBD dose administration periods.^8^ As with the article by Consroe et al.,^7^ no levels of parent THC were detected. These two articles seem to highlight the importance of testing for THC metabolites (i.e., 11-OH-THC and THC-COOH), for several hours or longer, as a means of documenting potential THC conversion after oral administration of CBD.

Taken together, the aforementioned studies seem to indicate a few trends. First, the majority of *a priori* empirical work on conversion of CBD to THC has *indeed* documented conversion in gastric fluid. Second, it appears that detection of conversion to THC in mammalian plasma should focus on THC metabolites (i.e., 11-OH-THC and THC-COOH) rather than solely measuring parent THC. This is supported by clinical research showing that blood/plasma THC levels are substantially lower after oral consumption than inhaled or IV routes of administration, and do not mirror pharmacodynamic drug effects.^10–12^ Finally, findings suggest that studies of gastric CBD conversion should widen to include documented conversion to other cannabinoids as well as 9α-OH-HHC and 8α-OH-*iso*-HHC.

In their commentary, Grotenhermen et al. also reviewed articles to demonstrate that administration of CBD does not produce THC-like effects in humans; however, the majority of the cited studies were not designed to determine whether oral administration of CBD produced effects similar to THC. Even so, a number of the reviewed (and some not reviewed) studies documented somnolence,^13,14^ lethargy,^13^ fatigue,^13^ and poor motor and cognitive performance^15^ after administration of oral CBD, symptoms traditionally associated with THC.^16,17^ Beyond direct conversion to THC, another pathway for the administration of oral CBD to produce THC-like effects may stem from its conversion to 9α-OH-HHC and 8α-OH-*iso*-HHC. Indeed, Watanabe et al.^5^ replicated prior work by Wilson et al.^18^ among others by demonstrating that CBD conversion to HHC can produce THC-like effects, including catalepsy and hypothermia, in mice. As THC-like intoxication effects may not only be driven by the conversion of CBD to THC but also the conversion of CBD to 9α-OH-HHC and 8α-OH-*iso*-HHC, it appears that oral administration of CBD could lead to unwanted consequences through a number of pathways.

There is substantial promise in the development of CBD as a medicine. The current evidence indicates that gastric conversion of CBD to THC has been relatively consistently observed across multiple studies over the past half century; however, the circumstances in which this happens, and the subsequent clinical consequences, remain uncertain. Thus, it is imperative that we continue to explore this issue among larger (particularly human) samples through *a priori* studies of potential side effects, including prospective evaluation of symptoms commonly associated with THC that employ validated instruments. Furthermore, it seems necessary for studies of CBD conversion to obtain multiple end points beyond plasma drug levels (e.g., urine drug levels, physiological and psychological effects). With the growing public and clinical interest in the use of CBD among various patient populations (e.g., people with epilepsy), we believe it is paramount to conduct rigorous studies to fully understand CBD's gastric conversion and potential THC-like side effects following its oral administration.

## Abbreviations Used

CBD: cannabidiol
THC: tetrahydrocannabinol
